# A novel method for the creation of a patient-specific realistic model of hypertrophic cardiomyopathy for surgical myectomy simulation

**DOI:** 10.1186/s41205-026-00321-1

**Published:** 2026-04-18

**Authors:** Sylvana García-Rodríguez, Tomomi Komatsu, Timothy Guenther, Abbey Thiel, Bradley W. Bolling, Joshua Hermsen

**Affiliations:** 1 Radius Medical Image Analysis Laboratory, Department of Radiology, University of Wisconsin – Madison, Madison, USA; 2 Department of Food Science, University of Wisconsin-Madison, Madison, USA; 3 Department of Surgery, University of Wisconsin – Madison, Madison, USA

**Keywords:** 3D printing, Rapid prototyping, Hypertrophic cardiomyopathy, Septal myectomy, Simulation

## Abstract

**Background:**

The use of three-dimensional (3D) printing to produce patient-specific models for operative planning and rehearsal of septal myectomy for patients with hypertrophic cardiomyopathy has been previously reported. However, human tissue-like characteristics have not been fully attained. This report describes a novel method to produce patient-specific models with improved tissue characteristics.

**Methods:**

A computed tomography data set was used to create a patient-specific model of the anterolateral portion of the left ventricle and proximal aorta. This was a basis for a mold, which included silicone liners to form the ventricular cavity and aortic lumen while sustaining high temperatures of the tissue material. This consisted of a hydrocolloid-based mixture that was poured into the 3D printed mold, allowed to solidify, and de-molded. A simulated myectomy surgery was performed on the model. Texture analysis testing and color testing were carried out and results were compared to a previous iteration, where the tissue was directly printed, as well as to fresh porcine cardiac tissue.

**Results:**

The presented methodology produced a realistic patient-specific model that was used to successfully rehearse a myectomy surgery. Cohesiveness and springiness of the gel medium was not significantly different than the printed medium or porcine tissue. The cutting force and firmness of the new medium was significantly different and lower when compared to both the printed medium and porcine tissue. Subjectively, the gel medium was softer, less “flaky”, and more muscle-like than the printed medium, and in general was considered as a definitive improvement.

**Conclusion:**

This study demonstrated a novel fabrication method for a patient-specific hypertrophy cardiomyopathy model by means of a gelatin-based formulation. The resulting model offers a consistency that is subjectively closer to human tissue, including the cutting feel. The resulting realistic model can be important in the development and dissemination of structured and standardized medical training programs in myectomy surgery.

**Supplementary Information:**

The online version contains supplementary material available at 10.1186/s41205-026-00321-1.

## Introduction

Hypertrophic cardiomyopathy is present in one in every 500 people, and is one of the most common causes of death among young patients [1]. Extended septal myectomy is the first-line treatment for patients with symptomatic hypertrophic cardiomyopathy and left ventricular outflow tract obstruction despite optimal medical management [2–5]. Even though the operation does not involve suturing, anastomoses or reconstruction, it can be difficult to learn and teach given the limited ability for multiple persons to share the operative view, the variability in anatomy from patient to patient, and the operative risks including creation of ventricular septal defect, injury to the aortic or mitral valves, and injury to the conduction system [3]. Nonetheless, the benefits of septal myectomy have driven the need for surgical intervention in hypertrophic cardiomyopathy [5]. Thus, the demand for surgeons and health centers with this capability has increased over time. It has been shown that the surgical outcome is directly correlated to the volume of myectomy procedures and expertise [6]. We have previously shown the utility of simulated resections using patient-specific 3D models for training of surgeons in performing septal myectomy.

Simulation-based surgical training is immensely beneficial not only for patient safety, but also for the skill development of medical trainees, as they can rely on repetition, detailed instruction and technical supervision and assessment [7, 8]. Feedback from medical professionals with various experience levels highlights the benefits of three-dimensional (3D) printed models for improving the understanding of the anatomy, but also for acquiring increasing confidence in performing several procedures repeatedly [8]. One of the difficulties in simulation of cardiac myectomy is the achievement of a model made from tissue-like material in addition to patient-specific, anatomical accuracy. The latter can be provided by 3D printing, but the development of materials able to mimic human tissue is still a growing field, and they can also be costly. This is precisely one of the most important requirements for myectomy simulations, where not only is the compliance and elasticity of the material important, but also the similarity while cutting the “tissue”. Several researchers have developed 3D cardiac models [8–11], either by printing directly with elastic and flexible materials or by casting into a mold [12]. Yamada et al. [13] developed a PVA-based material that was poured into a mold for medical training of various procedures. Hermsen et al. printed a hypertrophic cardiomyopathy model by printing it directly with a proprietary material. Myectomy surgeries were successful; however, the material is not readily available, and the texture felt slightly tougher than human tissue [14, 15].

Konjac is a plant-based polysaccharide that forms a versatile gel that can be used as a base for various applications, including tissue models. Nishio et al. [16] used konjac powder as a base for a fluorescence-guided surgical simulation model for tumor resection. Konjac noodles have been used as a model for anastomosis [17] and microsurgical training for plastic surgery [18]. Likewise, konjac gels have been used for endoscopic submucosal dissection of gastrointestinal tissue [19]. Previous studies have demonstrated that konjac glucomannan exhibits a synergistic interaction with κ-carrageenan, resulting in improved gel strength, elasticity, and water-holding capacity [20]. Utilizing this combination, we developed a tissue-like gel that closely replicates the texture of human cardiac tissue for surgical training applications.

The purpose of this study was to develop a hypertrophic cardiomyopathy model with patient-specific anatomy and constructed from tissue-like material for simulation-based training in myectomy surgery. We utilized 3D printing technology and a konjac gel formulation and compared texture analysis test results to those from porcine tissue and to previous iterations [14, 15].

## Methods

### Anatomical model

**Fig. 1:**
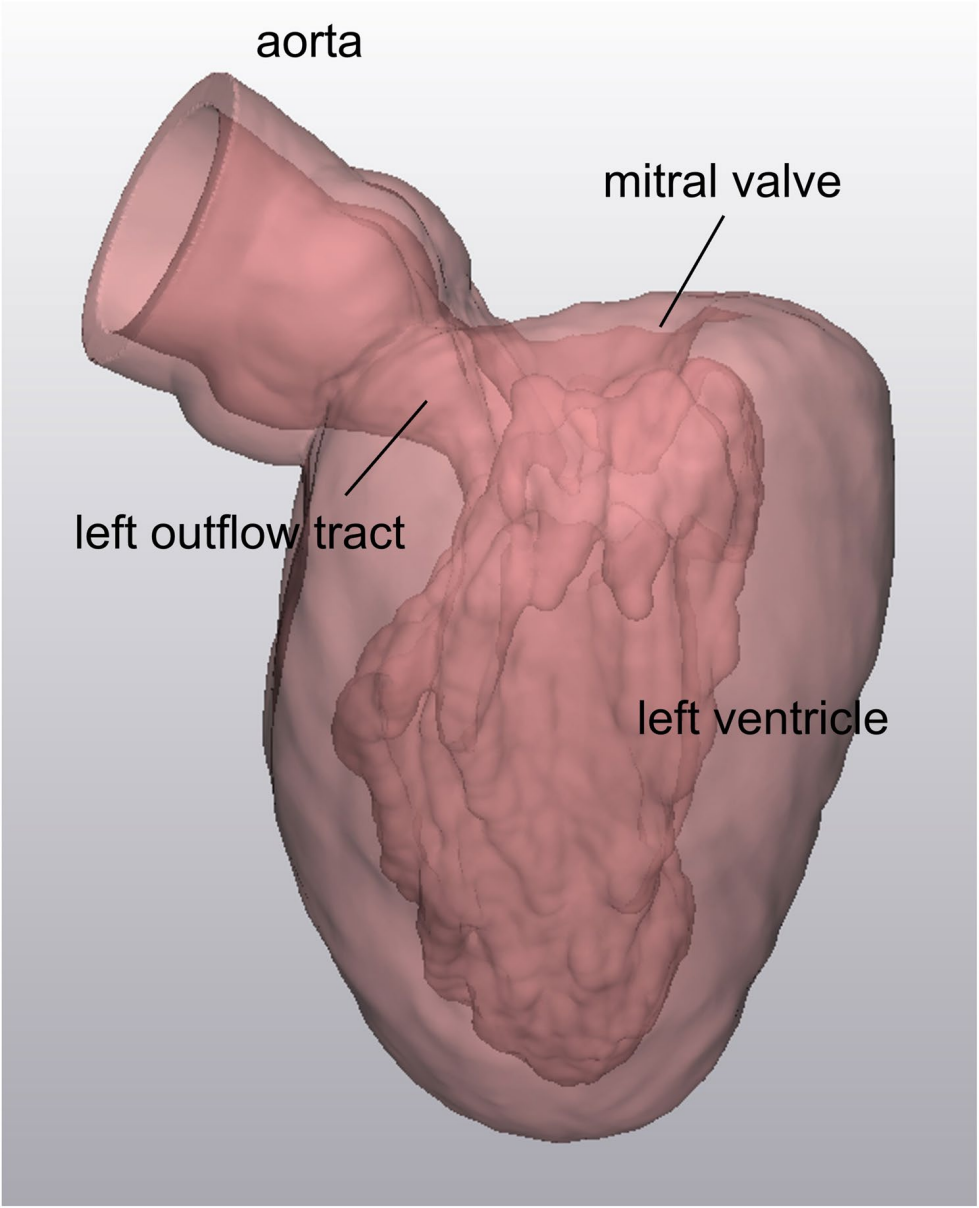
Virtual 3D model of hypertrophic cardiomyopathy anatomy of interest, showing the left ventricle, left outflow tract, mitral valve annulus and proximal aorta

Contrast-enhanced computed tomography images of an adult patient with hypertrophic cardiomyopathy were retrospectively obtained under IRB approval. This was a patient who had been operated upon years before, and was particularly chosen for this project given the high quality pre-operative CT imaging on file. It was also a fairly typical case of basal hypertrophy treated with standard trans-aortic extended myectomy. CT scanning (64 slice, Discovery CT 750 HD; GE Healthcare, Waukesha, WI) was prescribed within clinical routine for preoperative evaluation prior to septal myectomy, performed using prospective gating during mid-diastole. Scanning parameters included slice increment 0.625 mm, slice thickness 0.625 mm, 100 kvp, and current 500 mAs. Imaging data was imported and processed in Mimics (Materialise; Leuven, Belgium) to perform segmentation of the blood pool in the proximal aorta, proximal coronaries, and left ventricle, as well as the left ventricle myocardium. A virtual 3D model was generated (Fig. 1) and imported in 3-matic software (Materialise) for further processing, including surface defect corrections and smoothing. An artificial aortic wall was implemented and structures representing the blood pool were subtracted. Segmentation and virtual processing was performed by a Biomedical Engineer with extensive experience (approximately 10 years) in cardiac segmentation; segmentation accuracy was verified by a Radiologist.

### Mold design and fabrication

The 3D anatomic virtual model (Fig. 1) was used as the basis of a mold to cast a tissue-mimicking gel. Certain requirements and conditions were to be considered during the design phase: (1) the temperature of the gel preparation was 90 °C; (2) internal ventricular trabeculae conferred the geometry with significant complexity and the presence of undercuts, an obstacle in the de-molding process, and (3) the aorta and mitral valve annulus were to be kept circumferentially intact. The use of a 3D printed dissolvable core to obtain hollow cavities was not a feasible option due to the possibility of deformation under the high temperature of the gel.

**Fig. 2:**
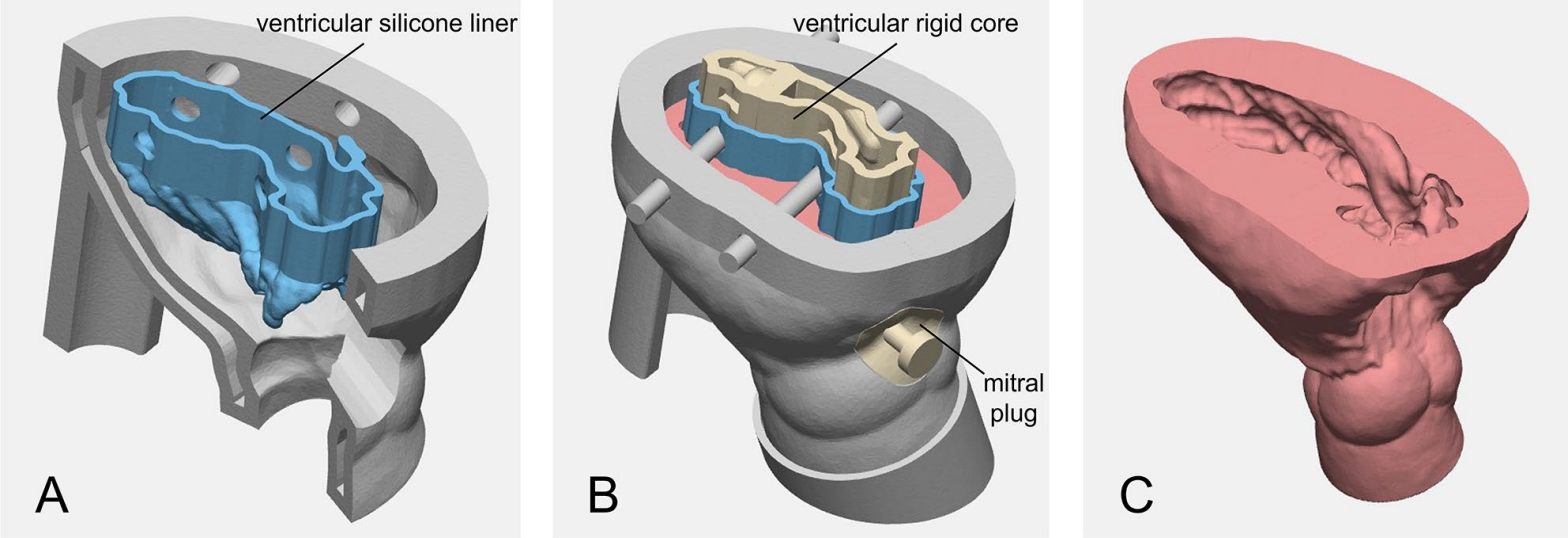
Cardiac mold design showing **A**) the outer mold shell and ventricular silicone liner with intricate inner wall features and trabeculae. The same concept was used to shape the aorta; **B**) the cardiac mold completely assembled and containing the konjac-based gel (pink). The aortic and ventricular silicon liners (blue, only ventricular silicone liner is shown) are in place and are kept from collapsing by means of rigid cores (yellow). The latter are connected internally by a peg and hole. The mitral valve annulus is shaped by the mitral plug (yellow), which is also connected with the ventricular rigid core by a peg and hole. Two rods across the ventricular silicone liner and rigid core keep the cavity structure at the correct anatomical position within the ventricle. **C**) Schematic of the resulting hypertrophic cardiomyopathy model, which contains the inner ventricular wall intricacies, aortic root geometry and mitral valve annulus, and consists of a single entity (no model assembly is required)

**Fig. 3:**
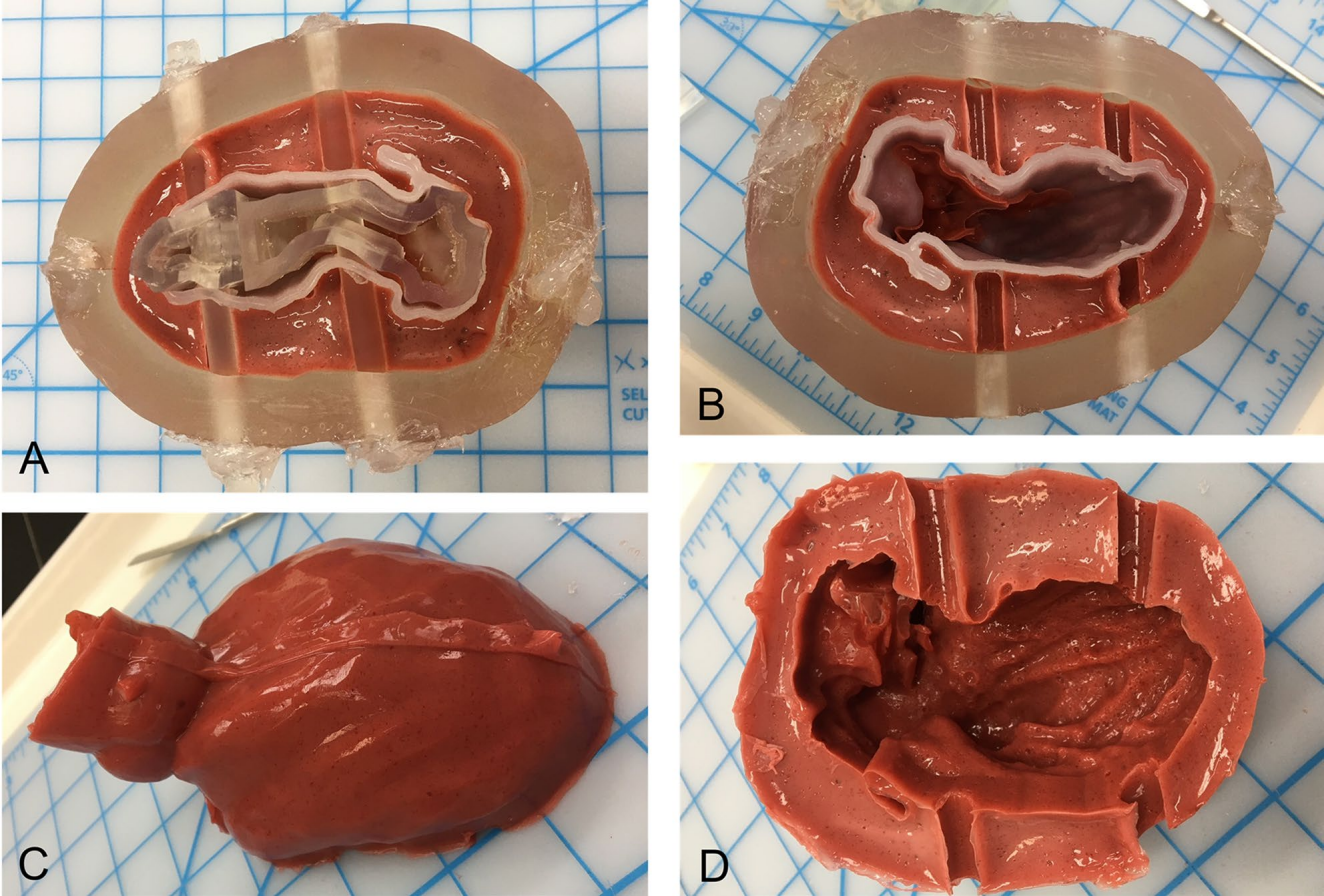
Photographs showing **A**) the completely assembled cardiac mold; **B**) the cardiac mold after the ventricular rigid core has been removed; **C**, **D**) resulting hypertrophic cardiomyopathy model showing the external surface as well as the internal ventricular wall and its intricacies in detail

To address these geometric issues, three separate elements were designed: a ventricular insert (Fig. 2A), an aortic insert, and a mitral plug (Fig. 2B). The latter consisted of a lid covering the mitral valve annulus while maintaining its anatomical geometry on the surface facing the ventricular lumen. The aortic and ventricular inserts were each composed of two parts: a silicone liner (Fig. 2A) and a core. Each silicone liner was fabricated with a separate 3D printed mold, with an average wall thickness of 2.5 mm. As the liners were designed to be in contact with the gel, they followed the inner wall geometry and surface features, such as trabeculae and coronary origins. A rigid 3D-printed core was inserted into each silicone liner to maintain its structure (Figs. 2B and 3A), especially during the pouring of the gel into the mold. The ventricular insert was kept in place by means of two rods across the mold (Figs. 2B and 3A). For de-molding, the rigid cores were removed (Fig. 3B), allowing the silicone liners to collapse for extraction from the aortic and ventricular lumens. A schematic and photograph of the resulting model are shown in Figs. 2C and 3C and D, where inner ventricular wall and surface intricacies are demonstrated.

All mold components were required to be impermeable; therefore, SLA was chosen as the 3D printing technique, which, in addition, offered high thermal stability and dimensional accuracy. The main mold was 3D printed (Form 3; Formlabs, Inc., Somerville, MA) using High Temp resin (Formlabs). The ventricular insert mold, aortic insert mold and mitral plug were 3D printed using clear resin (Formlabs). Liquid silicone (Ecoflex 00–30; Smooth-On, Inc., Macungie, PA) was cast into the molds, allowed to cure for approximately 24 h, and de-molded to obtain the ventricular and aortic silicone liners.

### Tissue-like gel preparation

Red and blue colorants, as well as a proprietary white titanium dioxide solution, were added to de-ionized water under stirring and heating. The aqueous solution was titrated with a potassium hydroxide solution (ACS grade; Fisher Scientific, Fair Lawn, NJ) using a micropipette until a pH of 10 ± 0.25 was achieved. A dry mix of κ-carrageenan (KC) and konjac glucomannan (KGM) was gradually added throughout heating with continuous stirring. Once the temperature reached 90℃, the solution was immediately poured into the mold (Video 1), allowed to cool to room temperature, and refrigerated overnight to set. The following day, the model was removed from the disassembled mold (Video 1, Figs. 2C, D) and used for surgical simulation.

### Surgical procedure

Septal myectomy was performed on the model situated within a chest mannequin, with standard instrumentation, in an operating room as previously described (Video 2) [21]. The volume of resection was quantified by liquid displacement in a graduated cylinder filled with 20 mL of water. The surgeon has extensive hypertrophy cardiomyopathy experience, with > 50 myectomies performed.

### Texture analysis

A portion of the model (gel media) was sectioned in 1 cm cubes and analyzed for hardness, cohesiveness, springiness, cutting force and work to cut. For comparison, samples with the same dimensions were obtained from a previously printed patient-specific model (using a proprietary hydrogel formula) [14, 15] (printed media), and septal muscle from freshly slaughtered pigs (porcine tissue). Measurements were performed in duplicate on four replicates of each medium.

A TA.XT2 Texture Analyzer (Texture Technologies, Hamilton, MA) with Exponent Software Analysis (Stable Micro Systems, Surrey, UK) was used for all texture tests. A TA-30 A probe (Texture Technologies) was used for compression tests and programmed to a pre-test speed, test speed, and post-test speed of 1 cm/s, 0.5 cm/s, and 1 cm/s, respectively. One test for each specimen consisted of two successive compressions by means of the probe to a strain of 50%. Resulting force vs. time data was used to carry out texture profile analysis and determine hardness, cohesiveness, and springiness of the samples. Hardness, a measure of firmness, was defined as the maximum force of the first compression [g]. Cohesiveness is a measure of the strength of the internal bonds, and is usually presented as a ratio (Eq. 1) of the area under the second curve to that under the first curve. A high ratio is an indication of strong internal cohesiveness, whereas the opposite is descriptive of a material that crumbles and breaks apart. Cohesiveness measures how well the sample withstands a second deformation relative to its resistance under the first deformation, and is defined as:

1 $$\:\mathrm{c}\mathrm{o}\mathrm{h}\mathrm{e}\mathrm{s}\mathrm{i}\mathrm{v}\mathrm{e}\mathrm{n}\mathrm{e}\mathrm{s}\mathrm{s}=\:\frac{{\mathrm{A}}_{\mathrm{p},\:2}}{{\mathrm{A}}_{\mathrm{p},\:1}}$$

where A_p, 1_ is the area under the positive force with respect to time from the first compression, and A_p, 2_ is the analogous area from the second compression. Springiness is defined as the elasticity or the ability of the medium to spring back to its original shape after it has been deformed during the first compression, and is expressed as the ratio:

2 $$\:\mathrm{s}\mathrm{p}\mathrm{r}\mathrm{i}\mathrm{n}\mathrm{g}\mathrm{i}\mathrm{n}\mathrm{e}\mathrm{s}\mathrm{s}=\:\frac{{\mathrm{t}}_{\mathrm{i}\mathrm{n}\mathrm{c},\:2}}{{\mathrm{t}}_{\mathrm{i}\mathrm{n}\mathrm{c},\:1}}$$

where t_inc, 1_ is the time during which the force is increasing in the first compression and t_inc, 2_ is the time of increasing force from the second compression.

For cutting tests, a TA-44 probe (Texture Technologies) equipped with a razor blade was used to slice into each sample. The probe was programmed to a pre-test speed of 1 cm/s, a test speed of 0.5 cm/s, and a post-test speed of 1 cm/s. The razor blade cut into the samples until a strain of 90% was attained. For all cutting tests, the cutting force and work to cut was determined. The first is defined as the force required to cut 90% through a sample using a scalpel; work to cut is the total work required to cut 90% through a sample and is recorded as the area under the curve (force with respect to time) during the cutting test.

All the gel and printed cubic samples were placed into a HunterLab ColorFlex EZ to analyze color, obtaining L*, a*, and b* values, where L* is indicative of lightness and a* and b* refer to the tendency toward red/green and yellow/blue, respectively. Each batch was analyzed in triplicate; means ± standard deviations were compared by two-tailed t-test.

## Results

The model fabricated with the konjac-based tissue-like gel was used in a simulated myectomy surgery and tested for texture for comparison with a directly printed media from a previous iteration and to porcine tissue. The surgery was successfully performed, where some subjective observations were noted by the surgeon. First, the model presented a wet consistency and a “flimsy” behavior, which is typical of heart structures in the absence of blood. The material was able to sustain a small suture and pulling from the suture, as well as stretching as if widening the aortic lumen (Video 2). Similarly, the material tolerated being held with surgical tweezers without crumbling, including both the myocardium and the resected tissue specimens. The cutting feel was satisfactorily similar to that of in vivo tissue.

From CT images, the design and fabrication process took approximately 3 weeks, including anatomy segmentation, mold design, and fabrication. Some of this time corresponds to printing and silicone curing. Complete assembly of the mold took approximately 30 min, and the gel pouring process and de-molding take approximately 30 and 40 min, respectively. Preparation of the tissue-like gel takes 40 min, including the time that it takes to heat. The cost of materials and equipment usage was approximately $300. This calculation was based on a one-year depreciation of the Form 3 printer, and does not include labor and laboratory supplies such as alcohol. The cost of the gelling agents and colorants totaled approximately $2 for one model.

**Fig. 4:**
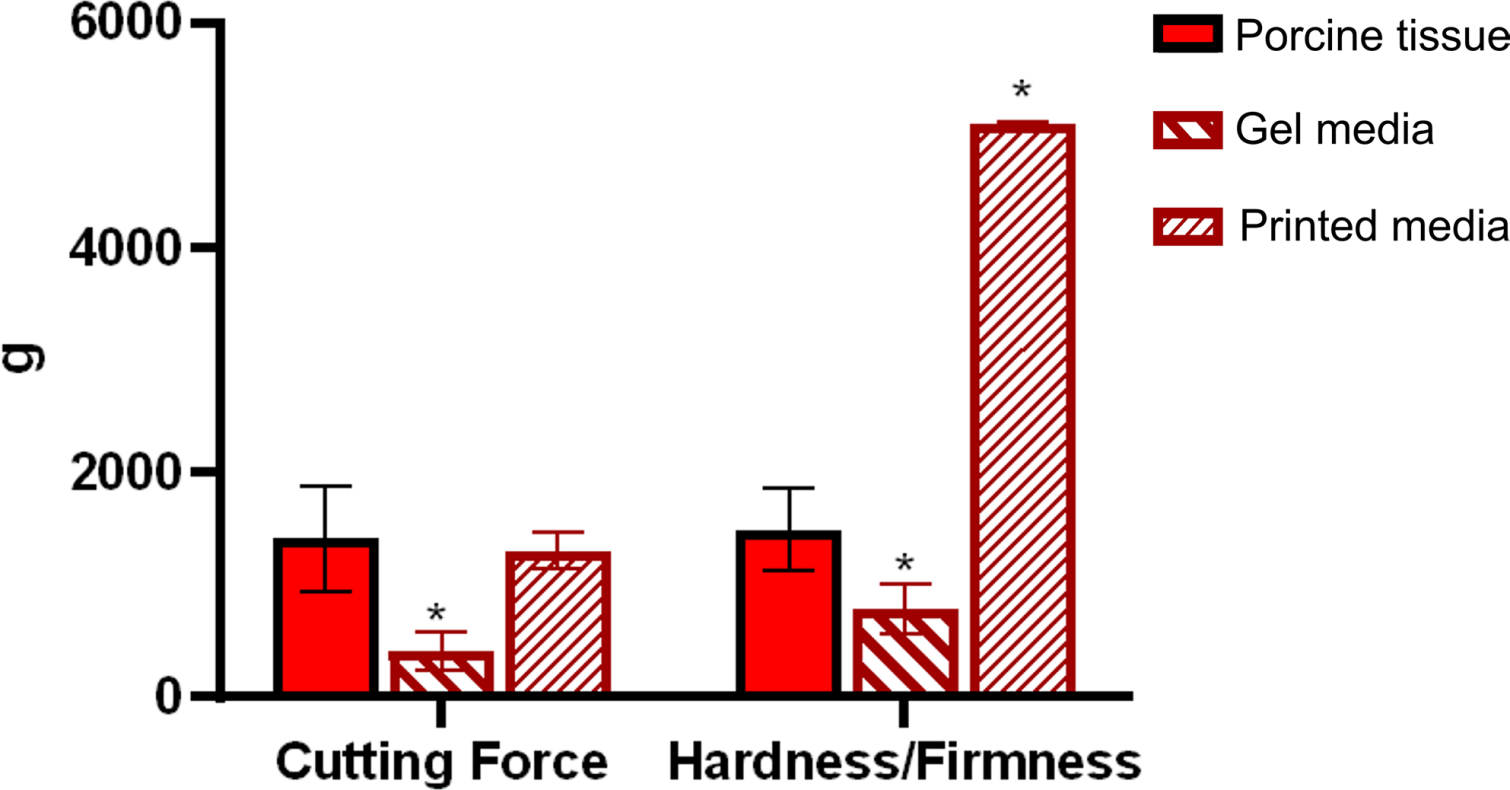
Texture analysis results, showing a comparison of porcine tissue, gel and printed media. The cutting force and hardness of the gel were significantly different from those of the other materials. However, the gel hardness was closer to porcine tissue than that of the printed media

**Fig. 5:**
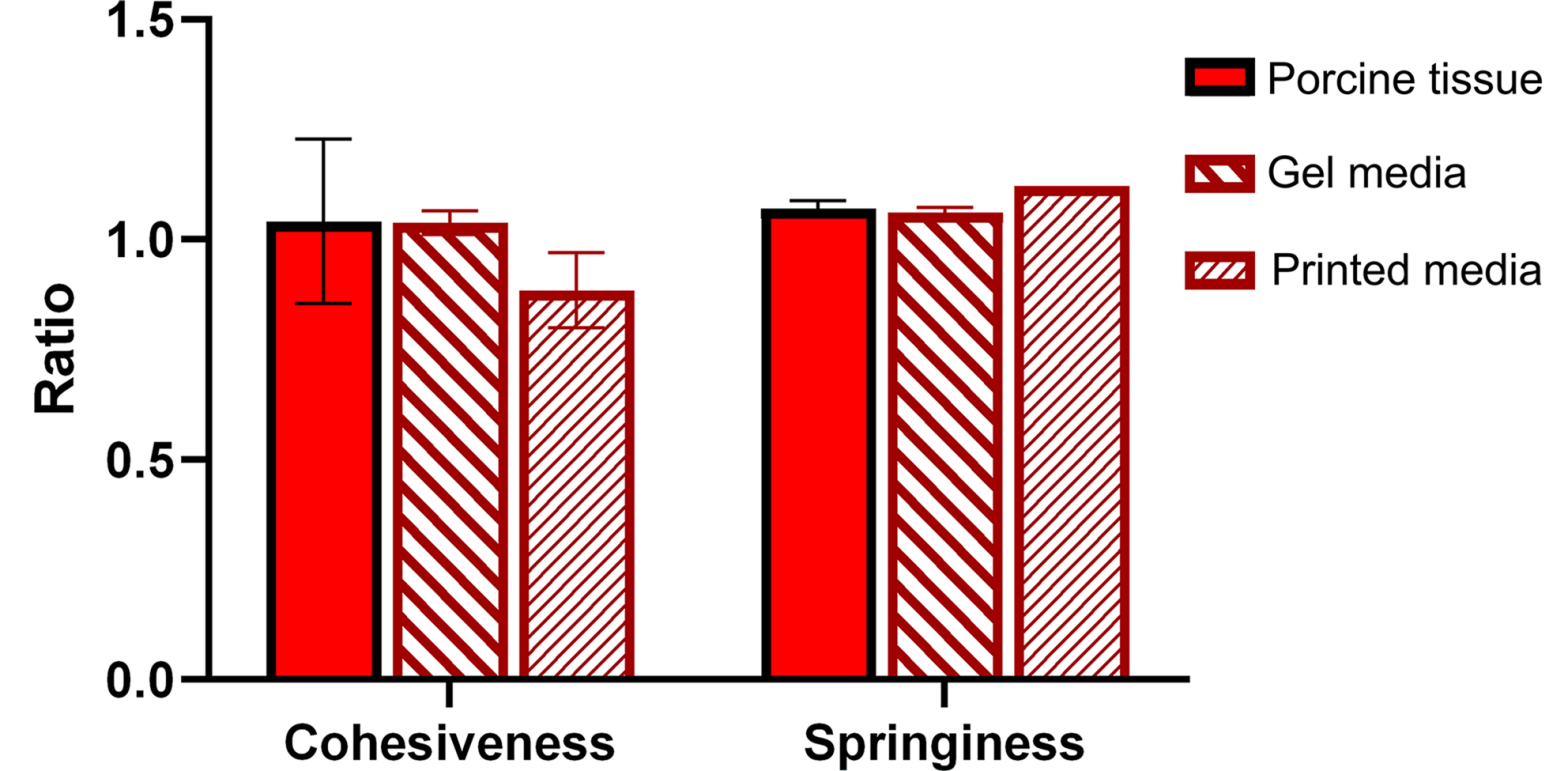
Results from texture analysis, showing that the cohesiveness and springiness of the gel media was similar to that of porcine tissue, slightly more than the printed media

The measure of hardness/firmness was 789 ± 217 g, 5109 ± 18 g, and 1491 ± 367 g, for the gel media, printed media and porcine tissue, respectively (Fig. 4). The cohesiveness ratio was 1.04 ± 0.03 for the gel media, 0.88 ± 0.09 for the printed media, and 1.04 ± 0.19 for the porcine tissue. The springiness ratio was 1.06 ± 0.013 for the gel media, 1.12 ± 0.0002 for the printed media, and 1.07 ± 0.18 for the porcine tissue (Fig. 5).

The mean cutting force was 410 ± 169 g for the gel media, 1308 ± 154 g for the printed media, and 1414 ± 470 g for the porcine tissue (Fig. 4). The work to cut was 550 ± 201 g*s, 102 ± 42 g*s, and 769 ± 85 g*s respectively.

The colorimetric analysis showed the following values for the gel media: L = 20.95 ± 2.07, a = 24.53 ± 0.74, b = 12.88 ± 0.07. In the case of the printed media, these results were: L = 37.8 ± 4.92, a = 17.59 ± 8.29, b = -6.05 ± 2.81.

A 2-way ANOVA test (α = 0.05) was used to compare physical properties between the porcine tissue and the printed and gel media. The hardness was statistically significantly different when comparing both media to porcine tissue (porcine vs. gel media, *p* = 0.007; porcine vs. printed media, *p* < 0.0001). There was no statistically significant difference in cohesiveness and springiness ratio when comparing gel and printed media with respect to porcine tissue (*p* > 0.05). Cutting force differed significantly between porcine tissue and gel media (*p* = 0.0003), but not between porcine tissue and printed media. The work to cut followed a similar pattern, but the difference between porcine tissue and gel media did not reach statistical significance (*p* = 0.08).

The color comparisons of the gel versus the printed media showed statistically different comparisons by two-tailed t-test across all three values L, a, and b (*p* < 0.05).

## Discussion

The use of patient-specific models for operative rehearsal and teaching of the extended septal myectomy operation has been previously described [14, 21]. Replication and expanded use of this technology has been hampered by site-specific factors, inaccessible materials, and limitations in rapid prototyping methodologies. Based on previous publications [14, 15, 21] and aiming to improve upon the physical characteristics of the model, we sought to re-engineer the process to produce a model that could be used to rehearse septal myectomy. We hypothesized that 3D printing a patient-specific heart mold would allow greater flexibility in experimenting with different and simpler materials that could be poured into the mold. Through an iterative process, the surgeon manipulated and cut different gel samples using a scalpel, resulting in a subjectively acceptable formulation of the hydrocolloid-based material described above. This also included achieving the optimal colorant combination to achieve a hue more closely approximating cardiac muscle (the previous printed models ranged from white to bright pink). The gel preparation incurred an additional constraint on the mold regarding the pouring temperature.

The mold provided a patient-specific geometry containing the proximal aorta, the left ventricular outflow tract, and the anterolateral left ventricular walls, while allowing its operation under high temperature. Several 3D printed cardiac models have been published previously [8–12]. However, room temperature conditions allowed for direct printing or the use of dissolvable printing materials to generate hollow anatomical structures, or printed the model using systems where the support material could be removed from the internal cavities. One example of a common dissolvable support material is polyvinyl alcohol (PVA), which is sensitive to temperature. This study shows an alternative fabrication process where patient-specific silicone liners are both resistant to high temperatures and collapsible for de-molding (Video 1). One advantage of our methodology was that the resulting model consists of a single entity; no assembly of anatomical parts was required, as is the case for some publications [12]. Despite the amount of mold pieces produced, the main mold and the silicone liners are reusable, thus allowing the fabrication of several model replicates.

Texture analysis results showed a lower hardness of the gel medium compared to both the printed medium and the porcine tissue, with a smaller difference when comparing the konjac gel to porcine tissue. Even though there was no significant difference when comparing both gel and printed materials to porcine tissue, the gel medium had a closer cohesiveness and springiness ratios to porcine tissue. These findings were reflected in the subjective input of an experienced surgeon after performing a myectomy on this model, where the konjac gel was perceived as softer and more cohesive or less “flaky” when compared to previous iterations. The force and work required to cut or incise the gel model were less than those for cutting the printed model and also less than those for cutting porcine heart tissue. However, the porcine tissue might demonstrate some differences due to a differing species [22] or to ex vivo conditions.

The surgical methodology of systematically carving the initial, usually dominant, piece of muscle by creating successively deep, attached “leaves”, was able to be replicated with a high degree of fidelity with the konjac-based model (Video 2). The konjac gel model was more akin to an empty, “deflated” heart encountered during surgery when compared to previously used printed models. Surprisingly, the resected specimens of the gel medium also demonstrated a degree of visual striation. Several of these described characteristics are not exhibited by other commonly used materials, such as silicone or elastic printing materials. Thus, the model resulting from this study is considered as a general improvement from past publications [14, 15].

The material properties of human myocardium encountered in septal reduction surgery are quite variable. The methods described in this study allow for the ability to vary the material properties of the model by modifying the konjac gel formulation. The synergistic interaction between KGM and KC influences the rheological and textural properties of the resulting mixed gels [23, 24]. KGM promotes the coil-to-helix transition of KC molecules, likely driven by an entropy increment provided by the KGM chains [23]. KGM molecules wind around the KC double helices via hydrogen bonding, which stabilizes the junction zones and fosters the formation of a dense, ordered three-dimensional network [25]. Under alkaline conditions, the deacetylation of KGM further enhances the mechanical strength and thermal stability of the composite system by enabling the formation of a thermally irreversible network through extensive intermolecular hydrogen bonding and hydrophobic interactions [25, 26]. The observed increase in gel hardness with higher KC proportions (e.g., 4.8 g KC to 3.2 g KGM) is attributed to the increased density of these rigid junction zones [27]. Conversely, a higher ratio of KGM (e.g., 3.2 g KC to 4.8 g KGM) leads to a softer texture, as the flexible KGM chains increase the elasticity of the matrix but dilute the rigid KC backbone [28, 29]. Based on this analysis, the next steps of this study include the variation of gel properties; models with different material properties can then be fabricated by re-utilizing the existing mold. This material experimentation is not easily feasible with print materials. Variation of the gel formulation may provide insights into the feasibility of developing models for other types of pathologies, surgeries, and tissues.

One limitation of this study is that it is comprised of the assessment of a single model, with the participation of a single experienced surgeon, who formulated a series of subjective observations. Future steps include a systematic and formal evaluation involving the input and feedback of several surgeons, by means of qualitative statistical survey-based assessments, of the model’s utility as an educational tool for teaching surgical myectomy. Similarly, we plan on incorporating medical trainees to qualitatively evaluate the contribution of the model as a tool for learning. We hypothesize that a material that more closely approximates human tissue may enhance the fidelity of the experience and more optimally prepare a trainee to perform a myectomy procedure.

The complexity of the mold may make it time-consuming to create models based on several patient’s anatomies. However, some simplifications can be implemented in the future, where fewer parts are needed for assembly. Some trials are also underway to explore if Formlabs clear resin can be used for the main mold, decreasing its cost and complexity. The development of improved printing technologies, such as the Formlabs Form 4 printer, will allow a shorter fabrication time, therefore decreasing its cost. Nonetheless, the ability to reuse the mold to produce multiple replicates of the model may be advantageous for an educational program where several trainees are evaluated using the same model, or for a single trainee that wants to practice an operation more than once. The low cost of materials to produce the tissue-like gel is advantageous, where one mold and several replicates will be less costly than printing several copies of a model. Five replicated models may cost approximately $310 for materials and printer usage, while producing several prints of one heart may be substantially more costly.

## Conclusions

In summary, the novel methodology presented here produces an operable patient-specific model fabricated with a tissue-mimicking material. This was achieved through a konjac-based gel formulation that can be poured into a patient-specific 3D-printed mold. The design and fabrication of the mold allowed high-temperature casting of the tissue-like gel. Incorporating silicone liners enabled the production of a patient-specific heart model with intricate surface structures that consisted of a single entity, without requiring assembly. Some texture characteristics were similar to those of porcine tissue, such as hardness and cohesiveness, when compared to a previous study involving a printed model using a custom material formulation. The mold is reusable, and the tissue-like gel ingredients are readily available and can potentially be varied to modify its material properties. The hypertrophic myectomy model presented here provides a valuable surgical training tool, and can offer insights into model design and tissue-like material development for various pathologies, tissues, surgical procedures, and several other applications in medical education.

## Supplementary Information

Below is the link to the electronic supplementary material.

Supplementary Material 1: Video 1: The tissue-like gel is poured in the cardiac mold immediately after heating. Once the gel is solidified, the ventricular and aortic rigid cores are removed, allowing the silicone liners to be collapsed and extracted. The model is then de-molded with minimal adhesion to the cardiac mold wall.

Supplementary Material 2: Video 2: A septal myectomy surgery was simulated using the tissue-like, konjac-based hypertrophic cardiomyopathy model. The behavior of the material was satisfactory, with a “flimsy” consistency, no crumbling, and “cutting feel” similar to that of tissue.

Supplementary Material 3

Supplementary Material 4

## Data Availability

Data sets generated during this study have been included as supplementary files.

## Abbreviations

3D: three-dimensional
KGM: konjac glucomannan
KC: κ-carrageenan
